# Types of Late-Life Challenges to Aging in Place

**DOI:** 10.1093/geroni/igab046.2145

**Published:** 2021-12-17

**Authors:** Kyeongmo Kim

**Affiliations:** Virginia Commonwealth University, Henrico, Virginia, United States

## Abstract

Many older adults prefer to live within their community because they have built strong relationships with their neighbors and neighborhood. Although housing-related factors promote aging in place, findings on the relationships of late-life challenges to aging in place (e.g., cost of living, autonomy) and relocation are mixed. Less is known about the types of challenges to aging in place and about the relationship between the types of challenges and relocation. Using data from the AARP 2015 Age-Friendly Community Surveys (N=3,190 adults aged 65 and older), this study examined the intersection of challenges to aging in place (e.g., home size, cost, safety, independence, family, transportation) and relocation (i.e., move to a different home outside of their community). Using latent class analysis (LCA), we identified five subgroups of late-life challenges to aging in place: multifaceted challenges, cost of living, independence, social connection, no concern. Findings from LCA with a distal outcome showed that older adults with multifaceted challenges (b=0.77, p<.001), were more likely to move out of their community, compared to those with lower levels of challenges, even after adjusting for age, sex, education, income, and chronic diseases. Also, those with challenges regarding the cost of living (b=0.84, p<.001), independence (b=0.64, p<.001), and family connection (b=0.45, p<.001) were more likely to expect to move out of their community. The findings highlight that older adults have different types of challenges to aging in place. Practitioners and policymakers should provide more individualized supportive services, considering the types of challenges to promote aging in place.

